# Comparative Analysis of Runs of Homozygosity Islands in Indigenous and Commercial Chickens Revealed Candidate Loci for Disease Resistance and Production Traits

**DOI:** 10.1002/vms3.70074

**Published:** 2024-12-10

**Authors:** Elaheh Rostamzadeh Mahdabi, Ali Esmailizadeh, Jianlin Han, Ming‐Shan Wang

**Affiliations:** ^1^ Department of Animal Science Faculty of Agriculture Shahid Bahonar University of Kerman Kerman Iran; ^2^ Key Laboratory of Genetic Evolution & Animal Models State Key Laboratory of Genetic Resources and Evolution, Kunming Institute of Zoology, Yunnan Laboratory of Molecular Biology of Domestic Animals, Kunming Institute of Zoology Chinese Academy of Sciences Kunming Yunnan China; ^3^ CAAS‐ILRI Joint Laboratory on Livestock and Forage Genetic Resources Institute of Animal Science Chinese Academy of Agricultural Sciences (CAAS) Beijing China

**Keywords:** chicken, disease resistance, genomic inbreeding, indigenous ecotypes, ROH islands, selection signature

## Abstract

Runs of homozygosity (ROH) are contiguous stretches of identical genomic regions inherited from both parents. Assessment of ROH in livestock species contributes significantly to our understanding of genetic health, population genetic structure, selective pressure and conservation efforts. In this study, whole genome re‐sequencing data from 140 birds of 10 Iranian indigenous chicken ecotypes, 3 commercial chicken breeds and 1 red junglefowl (RJF) population were used to investigate their population genetic structure, ROH characteristics (length and frequency) and genomic inbreeding coefficients (*F*_ROH_). Additionally, we examined ROH islands for selection footprints in the indigenous chicken populations. Our results revealed distinct genetic backgrounds, among which the White Leghorn breed exhibited the greatest genetic distance from other populations, while the gamecock populations formed a separate cluster. We observed significant differences in ROH characteristics, in which the commercial breeds showed a higher number of ROH compared to indigenous chickens and red junglefowls. Short ROH ranging from 0.1 to 1 Mb were dominant among the populations. The Arian line had the highest mean length of ROH, while the White Leghorn breed showed the highest number of ROH. Among indigenous chickens, the Lari‐Afghani ecotype exhibited the highest *F*_ROH_, but the Sari inherited the richest genetic diversity. Interestingly, GGA16 carried no ROH in the red junglefowls, whereas GGA22 had the highest *F*_ROH_ across all populations, except in the Isfahan ecotype. We also identified ROH islands associated with genetic adaptations in indigenous ecotypes. These islands harboured immune‐related genes contributing to disease resistance (*TLR2*, *TICAM1*, *IL22RA1*, *NOS2*, *CCL20* and *IFNLR1*), heat tolerance and oxidative stress response (*NFKB1*, *HSF4*, *OSGIN1* and *BDNF*), and muscle development, lipid metabolism and reproduction (*MEOX2*, *CEBPB*, *CDS2* and *GnRH‐I*). Overall, this study highlights the genetic potential of indigenous chickens to survive and adapt to their respective environments.

## Graphical abstract

There are distinct genetic backgrounds among different chicken populations, with commercial breeds showing a higher number of runs of homozygosity compared to indigenous chickens and their wild ancestors.

Indigenous chicken ecotypes have runs of homozygosity islands associated with genetic adaptations, including genes related to disease resistance, heat tolerance, oxidative stress response and muscle development.

The White Leghorn breed exhibited the greatest genetic distance from other populations, while gamecock populations formed a separate cluster

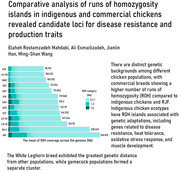

## 1 Introduction

The increase in the global human population and the subsequent rise in demand for animal‐sourced foods, coupled with the challenges posed by climate change, have highlighted the need for resilient and environmentally compatible livestock species. In this context, indigenous chicken breeds and ecotypes are being recognised and considered for their potential to address these challenges. Breeds are a distinct group of domestic livestock with a shared history, resulting from deliberate human breeding practices to develop specific characteristics. On the other hand, ecotypes are a subpopulation within a breed, shaped by natural selection in response to local environmental conditions (FAO 2013). Indigenous chicken ecotypes often exhibit higher genetic diversity compared to breeds. Furthermore, they have evolved traits that enable them to thrive in specific local conditions, including resistance to prevalent diseases and tolerance to harsh climatic conditions (Padhi 2016).

Indigenous chickens in Iran exhibit genetic diversity, local adaptation and cultural significance and play a vital role in supporting livelihoods and biodiversity conservation (Shahbazi, Mirhosseini, and Romanov 2007; Vali 2008). Despite facing challenges such as limited resources and veterinary care, these chickens thrive in their natural environment. Conservation initiatives are essential to preserve their distinctive genetic heritage. The identification of key genes related to disease resistance and heat stress tolerance can bolster their sustainability (Banos et al. 2020). By pinpointing genes associated with these traits, breeders can develop more resilient chicken breeds that maintain productivity in harsh climates.

The rapid technological advancements in DNA sequencing have greatly facilitated the availability of large‐scale whole genome sequencing data for in‐depth investigation of genomic patterns relevant to various biological applications (Reuter, Spacek, and Snyder 2015). Runs of homozygosity (ROH) are defined as stretches of the genome where an individual has inherited identical copies of chromosomal segments from both parents due to their recently shared ancestry (Ceballos, Hazelhurst, and Ramsay 2018). Factors such as inbreeding, population bottleneck, genetic drift and selection (natural or artificial) can contribute to the presence of ROH throughout the genome (Colpitts, McLoughlin, and Poissant 2022; Falconer and Mackay 1996; Hewett et al. 2023; Martin et al. 2023). It is important to note that the occurrence of ROH can have both positive and negative consequences. While ROH can be associated with detrimental effects such as increased risk of recessive genetic disorders, they can also be linked to positive traits due to the selective pressures acting on certain genomic regions (Pemberton et al. 2012). ROH have gained significant popularity in assessing both inbreeding level (*F*_ROH_) and identifying selection signatures (ROH islands) within livestock populations (Mastrangelo et al. 2017; Rostamzadeh Mahdabi et al. 2021; Zhang et al. 2018). The *F*_ROH_ is indeed suggested to be one of the most powerful methods for estimating inbreeding in a population (Matthew C Keller, Visscher, and Goddard 2011). Islands of ROH, which are genomic regions with an unusually high density of ROH, can indeed be indicative of selective pressure in a population (Kim et al. 2013). By examining ROH patterns in livestock populations, regions of the genome that have been under selection due to their association with desirable traits such as increased meat or milk production, disease resistance or adaptation to specific environments have been identified (Goli et al. 2024; Kim et al. 2013; Mastrangelo et al. 2017; Peripolli et al. 2018). These ROH islands can provide valuable insights into the genetic basis of important traits in livestock species.

This study aims to provide significant insights into the evolutionary history, genetic diversity and population genetic structure of indigenous Iranian chickens, commercial breeds, and RJF. By analysing whole genome re‐sequencing data, the distribution of ROH, within‐population genomic inbreeding and ROH islands across the chicken genomes was investigated to elucidate the genetic characteristics and adaptations of these chicken populations.

## 2 Materials and Methods

### 2.1 Sample Collection, Genotyping and SNP Calling

The indigenous chickens included in this study were collected from 10 different locations of Iran (Table S1, Figure S1), representing a diverse range of seven ecotypes (Shiraz (7), Sari (11), Mashhad (12), Isfahan (10), Zahedan (14), Tabriz (10) and Yazd (10)) and three gamecock populations (Iranian Lari (14), Pakistani Lari (7) and Afghani Lari (11)). Three commercial breeds (Arian (12), White Leghorn (11) and Cornish (1)) and a wild population of red junglefowl (10) were used for comparison. Genomic DNA was extracted from whole blood using the modified phenol‐chloroform protocol (Iranpur, Esmailizadeh, and Horriat 2010) and all samples were sequenced to a mean coverage of 8.02X (Table S1) (Wang et al. 2020).

The FastQC (Andrews 2010) and Trimmomatic (Bolger, Lohse, and Usadel 2014) software were applied for examining short reads and trimming adaptors and low‐quality base pairs, respectively. Alignment of the qualified reads to the chicken reference genome (GRCg6a) was carried out by using the Burrows‐Wheeler Aligner (BWA) software (Li and Durbin 2009). PCR duplicates were removed by Picard tools with default parameters (http://broadinstitute.github.io/picard/). Local realignment and base recalibration were performed using Realigner Target Creator, Indel Realigner, BaseRecalibrator and Print Reads arguments in the GATK software (v3.8) (McKenna et al. 2010). To call single nucleotide polymorphisms (SNPs), we utilised the Unified Genotyper argument in GATK. Subsequently, hard filtering was conducted using the ‘Variant Filtration’ argument with the following thresholds: QD < 2.0, FS > 60.0, MQ < 40.0, MQRankSum < −12.5, ReadPosRankSum < −8.0. For further analysis, we focused on SNPs located on autosomes and applied stringent quality control measures using PLINK v1.9 software (Purcell et al. 2007). To ensure data reliability, we excluded samples and SNPs with a call rate < 90%, as low‐quality DNA can lead to missing alleles and incorrect genotype calls during sequencing or genotyping (Barendse et al. 2009). Additionally, we removed SNPs exhibiting significant deviations from Hardy‐Weinberg equilibrium (HWE) with a *p*‐value < 0.000001. While deviations from HWE can sometimes be attributed to selection; they are more likely to result from technical issues. In WGS, it is generally assumed that deviations from HWE are the result of genotyping errors (Teo et al. 2007). Finally, we applied a minor allele frequency (MAF) < 0.05, focussing on SNPs with a greater chance of contributing to phenotypic variation. MAF filtering is typically carried out under the assumption that singletons or other rare variants, occurring at a frequency of less than 5% within a sample group, are likely to be attributed to genotyping errors (Hemstrom et al. 2024).

The final dataset consisted of 14,709,478 autosomal SNPs. In order to reduce artefacts caused by LD (linkage disequilibrium), pruning SNPs with high r^2^ values was performed using (–indep‐pairwise 50 10 0.1) in the PLINK v1.9 (Purcell et al. 2007).

### 2.2 Population Genetic Structure

An overview of the population genetic structure was obtained using principal component analysis (PCA). The clustering of all populations was formed by the SNP Relate package in R software (Wickham 2009; Zheng et al. 2012). Moreover, to investigate the admixture proportions (or ancestries) and infer ancestries, the approach based on maximum likelihood estimation between populations was applied using the ADMIXTURE software (v1.3.0) (Alexander, Novembre, and Lange 2009). To assess the accuracy of the admixture analysis, cross‐validation (CV) errors were calculated by running the ADMIXTURE with *K* = from 2 to 9.

### 2.3 Distribution of ROH Across the Genome

ROH segments were detected using the PLINK v1.9 with the following options: a minimum number of 50 SNPs to define an ROH (homozyg‐snp), up to 3 heterozygous markers allowed in a window (homozyg‐window‐het), a sliding window size of 100 kb (homozyg‐kb), a minimum density of 50 for considering a ROH (homozyg‐density), up to 5 missing calls permitted in a window (homozyg‐window‐missing), a window size of 50 SNPs (homozyg‐window‐snp), a gap size of 1000 kb between two SNPs (homozyg‐gap) and a proportion of homozygous overlapping windows threshold of 0.05 (homozyg‐window‐threshold). The mean number and length of ROH were estimated per autosome and per population. ROH segments were further categorised into length classes short (0.1–1 Mb), medium (1–2 Mb) and long (>2 Mb). The results of ROH analyses were visualised using the R package ‘detectRUNS’ (Biscarini et al. 2018).

The inbreeding coefficient based on ROH (*F*_ROH_) was estimated in different length classes of ROH. This estimation was performed according to the method introduced by McQuillan et al. (2008), which calculates the ratio between the total length of all ROH in each bird and the total size of autosomal SNP coverage.

To evaluate within‐population genetic diversity, we utilised the VCFtools v0.1.15 software (Danecek et al. 2011) to estimate the observed number of heterozygous sites by applying the ‘–het’ function.

### 2.4 Detection of Common ROH Across Populations

Consensus sequences of ROH in each population were provided using the ‘homozyg‐group’ parameter in the Plink v1.9. A selection criterion was then employed to identify ROH islands, which represented the genomic regions where overlapping ROH were shared by more than 70% of individuals within a population. These regions were considered highly homozygous, based on 95% identity in allelic matches to ensure their high similarities.

To annotate the detected genomic islands, we employed the Variant Effect Predictor (VEP) tool (http://asia.ensembl.org/info/docs/tools/vep/index.html). We also used the DAVID database (Database for Annotation Visualisation and Integrated Discovery) (https://david.ncifcrf.gov/home.jsp) for the enrichment of candidate genes. The results of gene annotation were statistically determined to be significant if the *p*‐value was less than 0.05 for the GO term (gene ontology) and KEGG (Kyoto Encyclopaedia of Genes and Genomes) pathways.

## 3 Results

### 3.1 Population Genetic Structure

**FIGURE 1:**
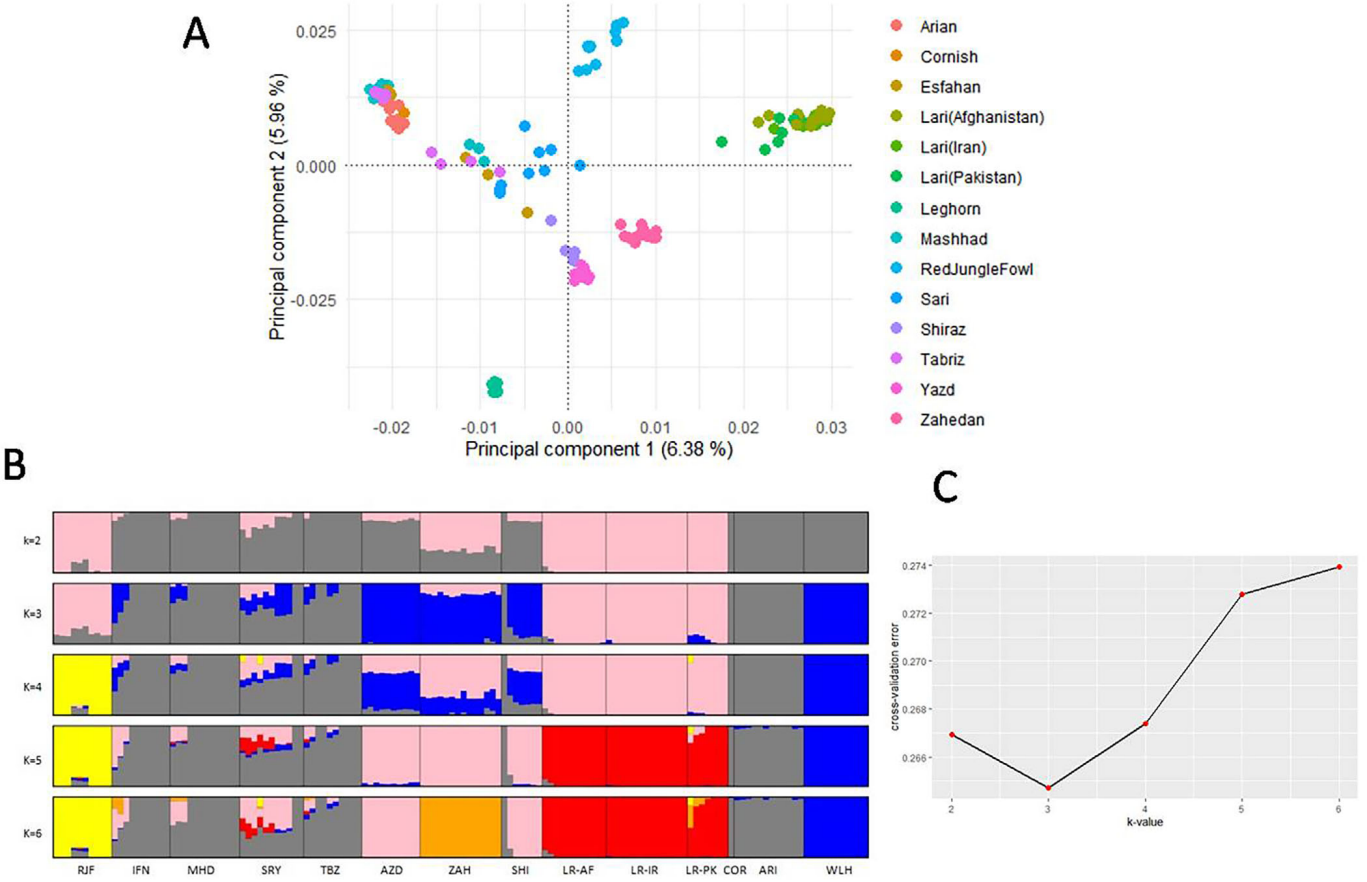
Population genetic structure. (A) Clustering of the chicken populations visualised by a PCA plot. (B) The genetic structure inferred from the admixture analysis of the different populations. (C) The plot of the cross‐validation errors as a function of different *K* values.

The first and second principal components disclosed distinct groupings among all samples, which were supported by the admixture analysis (Figure 1). The first principal component (PC1) accounted for 6.38% of the total variance to differentiate WLH from both indigenous ecotypes and RJF, highlighting a substantial genetic divergence of WLH. The PC2 explained 5.96% of the total variance (Figure 1A) to separate the ARI line from most of the populations, though it was relatively close to the IFN, MHD and TBZ populations primarily found in northern Iran. This suggested that intentional crossbreeding might have occurred between these three ecotypes and the ARI line to enhance meat production traits. The ZAH, SYZ, and AZD ecotypes distributed in southern Iran formed a distinct group but they exhibited close genetic proximity to each other.

At *K* = 2, all three commercial breeds were classified in one group, while all the Lari chickens and RJF were clustered in another group (Figure 1B). The remaining indigenous ecotypes showed mixed genetic components derived from these two groups. The CV error value was found to be the lowest at *K* = 3, indicating the presence of three potential ancestries (Figure 1C), which separated the WLH breed from the ARI and COR chickens, while AZD, ZAH and SYZ exhibited a shared ancestry with WLH. Similarly, TBZ, MHD, IFN and SRY displayed additional admixture with the Arian line. At *K* = 4, RJF exhibited a unique genetic makeup (yellow colour), independent from all the Lari chickens. At *K* = 6, there was a further subdivision within ZAH, while SYZ and AZD stood together.

### 3.2 Distribution of ROH Within the Populations and Throughout the Genome

We identified 53,857 ROH (an average of 508 ROH per bird) among all indigenous chickens, 18,945 ROH (an average of 824 ROH per bird) in the commercial breeds, and 4193 ROH among all RJF (an average of 419 ROH per bird) (Table S2). It is inappropriate to compare the absolute numbers of ROH across populations with different sample sizes, so to ensure a fair comparison, we focused on the average number of ROH per bird. As it was expected, we observed higher numbers of ROH in the commercial birds than in the indigenous chickens and RJF.

**FIGURE 2:**
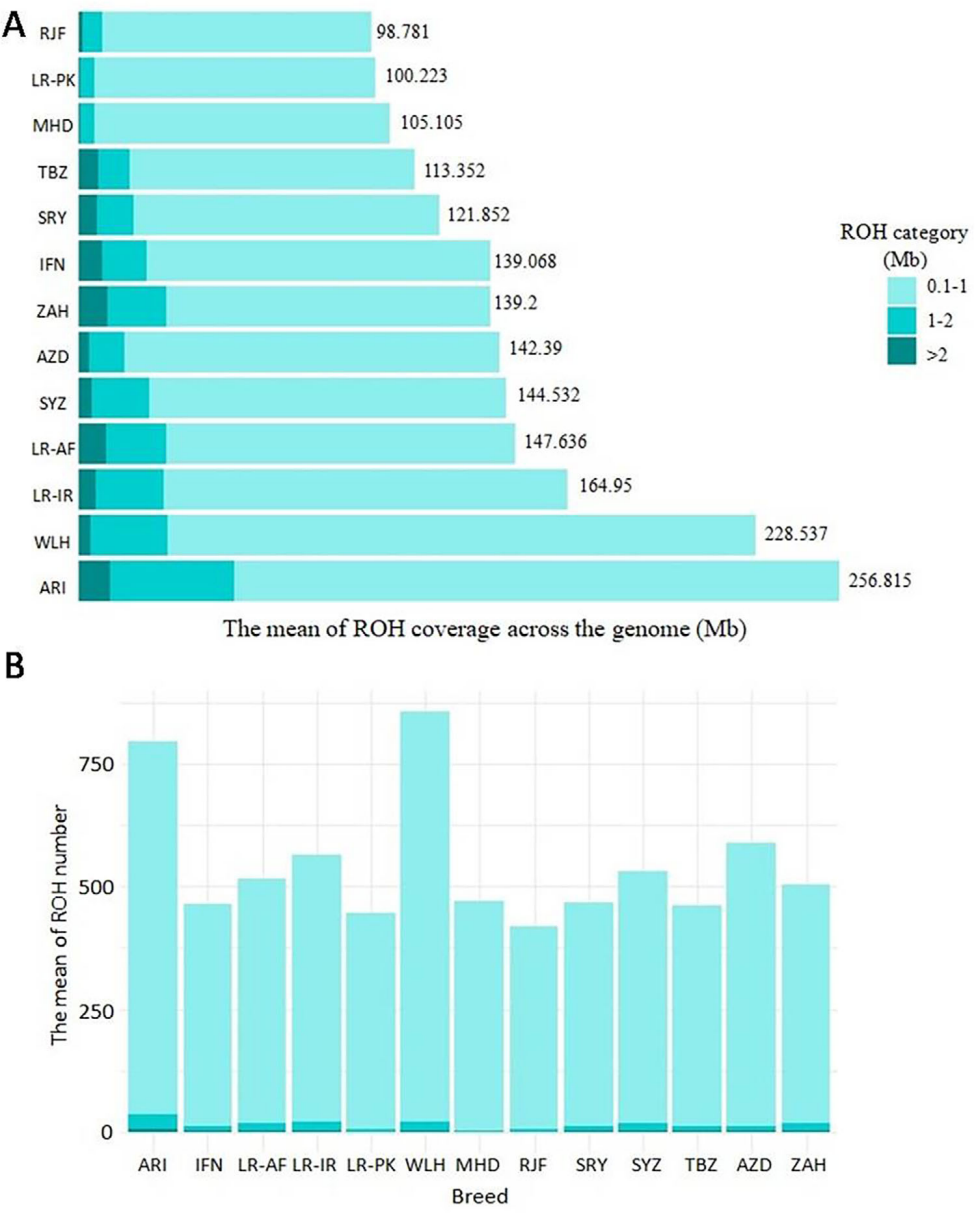
Comparative analysis of the ROH occurrence across the chicken genome in different populations. (A) The mean length of ROH and (B) the mean number of ROH per individual, categorised by the ROH length classes.

The ARI line had the longest mean ROH, covering 256 Mb of the genome, while the WLH line contained the highest number (834) of ROH. In contrast, the RJF population exhibited the shortest mean and lowest number (419) of ROH, covering only 98.78 Mb of the genome. Among the indigenous and gamecock chickens, the LR‐IR population had the longest genomic coverage by the ROH (164.95 Mb), while LR‐PK had the shortest genome coverage (100.223 Mb). Regarding the number of ROH, AZD had the highest number of ROH (587.7) but SRY displayed the lowest number of ROH (467.73) (Figure 2).

Furthermore, all chicken populations exhibited a relatively high number of short ROH segments between 0.1 and 1 Mb. The ARI line had a larger proportion of medium and long ROH (>1 Mb), totalling 52.7 Mb, which was the longest among all populations. Among the indigenous ecotypes, the highest proportion of ROH > 1 Mb was observed in ZAH, accounting for 29.76 Mb.

**FIGURE 3:**
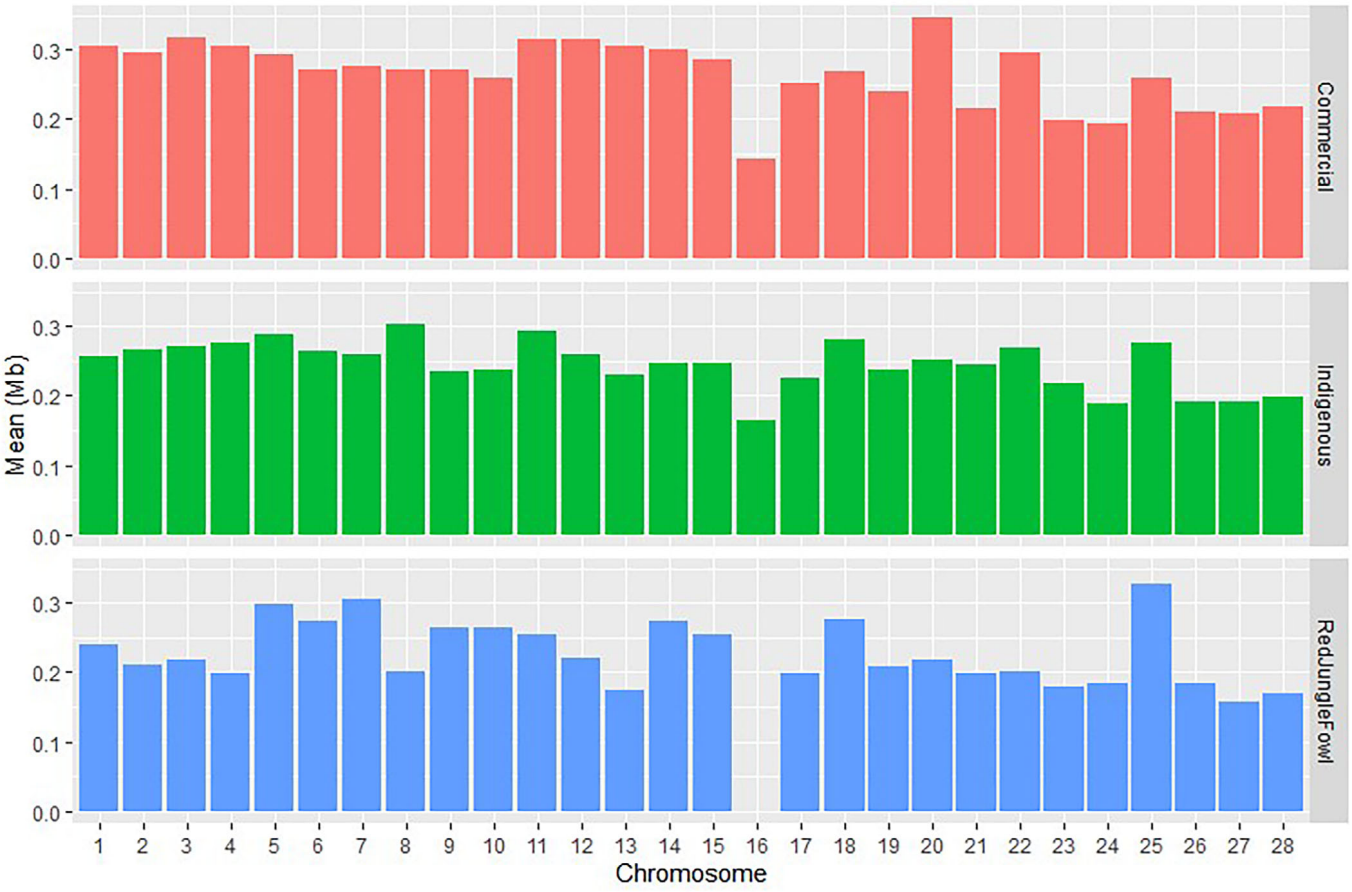
Mean ROH length per chromosome in the various chicken populations.

It was particularly interesting to note that GGA16 carried very few ROH across all populations, even to be absent in RJF. The indigenous populations showed the longest genomic coverage of ROH on their GGA8, while the commercial breeds had more ROH on their GGA20 and RJF exhibited the highest ROH coverage on their GGA25 (Figure 3).

### 3.3 Within‐Populations Genomic Inbreeding (*F*_ROH_)

**FIGURE 4:**
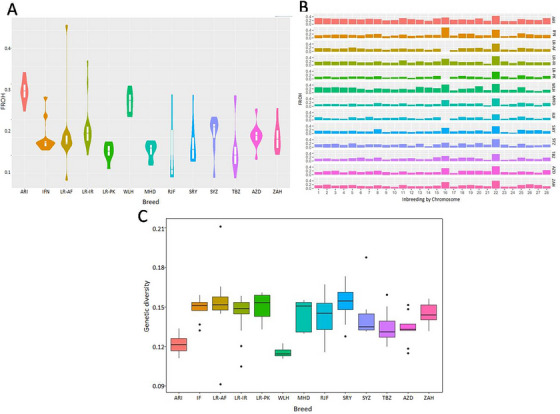
The genome‐wide distribution of genetic diversity in the 140 birds of the indigenous, commercial and red junglefowl populations. (A) *F*_ROH_ calculated for each population. (B) *F*_ROH_ calculated per chromosome within each population. (C) Nucleotide diversity values estimated for each population.

As anticipated, all the commercial birds demonstrated the highest average *F*_ROH_ (Figure 4A). The ARI and WLH lines indicated an average *F*_ROH_ of 0.279 and 0.248 per bird, respectively. Conversely, the lowest *F*_ROH_ was obtained in RJF (0.107). Among the indigenous and gamecock chickens, LR‐IR had the highest *F*_ROH_ (0.179) but LR‐PK exhibited the lowest *F*_ROH_ (0.109). Across different ROH classes, the highest *F*_ROH_ was found in the ROH between 0.1 and 1 Mb, followed by the ROH between 1 and 2 Mb and then the ROH > 2 Mb (Table S2). GGA22 exhibited the highest *F*_ROH_ in most populations, except for IFN, which carried the highest *F*_ROH_ on GGA16 (Figure 4B).

In terms of average genetic diversity, the SRY had the highest value of 0.153, while AZD presented the lowest value of 0.133. In contrast, the WLH and ARI lines had the lowest values of 0.115 and 0.121, respectively (Figure 4C).

### 3.4 Putative Signatures of Selection in ROH Islands

In our study, we considered ROH islands as selection signatures if they were present in more than 70% of chickens in a population and observed 190 to 312 of such islands in indigenous chickens (Table S3). As it was expected, the numbers of identified islands were higher in the WLH (801) and ARI (504) lines. The shared regions of ROH islands between at least two populations were found on GGA1, 2, 3, 4, 5, 7, 8, 10, 17, 18, 24 and 27 (Table 1). Four genes (*GFPT1*, *EBF2*, *LMX1B* and *S100B*) were common across all populations (Table S4) and they were associated with the development of adipose tissue (*EBF2*), organogenesis (*LMX1B*) and innate immune response (*S100B*). In addition, 13 genes (*SLC23A2*, *S100B*, *PPP2R2A*, *PCNA*, *OSGIN1*, *NFU1*, *LMX1B*, *GFPT1*, *FANCF*, *EBF2*, *DPYSL2*, *COL6A2*, *CDS2*, *C2CD2L* and *BNIP3L*) were common among indigenous chickens and they were involved in various biological functions such as lipid metabolism, pigmentation and response to hypoxic stress, etc.

**TABLE 1:** The ROH islands identified in at least two populations.

GGA	Start	End	ROH length	No. of chickens	Ecotype
1	104254119	104407047	152928	13	SYZ, RJF
1	24870769	24995809	125040	18	WLH, TBZ
1	52850106	53086867	236761	16	ARI, AZD
2	21759794	22233224	473430	21	TBZ, LR‐AF
2	36269154	36375734	106580	23	WLH, MHD
2	119603316	119708788	105472	17	WLH, LR‐PK
2	81744523	81844947	100424	20	LR‐IR, RJF
2	36269187	36375879	106692	20	IFN, RJF
2	32591290	32756351	165061	19	ARI, SYZ
3	13305233	13406423	101190	19	WLH, ARI
4	54678018	55034283	356265	17	AZD, SYZ, LR‐AF
4	54678018	55034283	356265	17	AZD, SYZ
4	82687	205506	122819	17	MHD, SYZ
5	46808365	47744054	935689	17	AZD, IFN
5	3573488	3682638	109150	25	ARI, ZAH
8	53274	206703	153429	24	ZAH, IFN
8	10872478	10974161	101683	14	WLH, SYZ
8	10576243	10694905	118662	16	LR‐PK, LR‐AF
10	140482	265674	125192	24	ARI‐MHD
17	5260217	5409401	149184	21	ZAH, TBZ
17	10168242	10283259	115017	18	ZAH, SYZ
18	7016657	7326895	310238	19	ARI, SYZ
24	3308	112441	109133	34	ZAH, IFN, LR‐IR
27	915627	1038132	122505	20	AZD, LR‐AF

In the indigenous chickens, out of 1248 identified genes, we observed significant enrichments (*p* < 0.05) in 61 biological processes (BP), 52 molecular functions (MF) and 34 cellular components (CC), as well as 19 KEGG pathways. For the commercial breeds, among the 424 detected genes, we found significant enrichments in 23 BP, 16 MF and 19 CC, along with 7 KEGG pathways. In the RJF population, out of 100 genes, we observed significant enrichments in 5 BP, 6 MF and 0 CC, as well as 1 KEGG pathway (Table S5).

Notably, several pathways associated with disease resistance, including defence response to virus (GO: 0051607), cytokine‐mediated signalling pathway (GO: 0019221), inflammatory response (GO: 0006954) and Influenza A (gga05164), were commonly detected across several populations. Additionally, the MAPK signalling pathway (gga04010) and the GnRH signalling pathway (gga04912) were identified as shared pathways among different populations of IFN, LR‐AF, SRY and TBZ. In MHD, the *KCNMA1*, *LOXL2*, *MMP2* and *NOS2* genes were found to be involved in response to hypoxia (GO: 0001666) (Table S5).

## 4 Discussion

To understand genetic relationships and identify regions of potential selection, we examined genetic diversity and ROH distribution across the genome in Iranian indigenous, commercial and red junglefowl chickens using whole genome re‐sequencing data.

### 4.1 Population Genetic Structure and Diversity

The domestic chicken (*Gallus gallus domesticus*) is believed to have originated from the red junglefowl (*Gallus gallus*), which is native to Southeast and South Asia, where other subspecies of RJF may have contributed to the genetic diversity of domestic chicken. (Wang et al. 2020).

PCA and admixture are distinct methods employed for analysing genetic data and inferring population structure. While both techniques strive to reveal patterns of genetic variation within a dataset, they can sometimes produce contrasting results that can be attributed to the different underlying assumptions and methodologies of the two approaches. The admixture analysis is often more adept at detecting recent or subtle admixture events, whereas PCA tends to emphasise broader patterns of genetic variation. PCA operates under the assumption of genetic similarity among individuals, whereas admixture characterises individuals as combinations of ancestral populations. Discrepancies in outcomes can arise from these differing assumptions, contributing to divergent results between the two methods (Novembre and Stephens 2008; Pickrell and Pritchard 2012).

Our PCA and admixture analyses indicated that the gamecock populations formed a distinct cluster separate from other indigenous breeds. This finding aligns with reports documented in the literature (Zhang et al. 2020; Zhi et al. 2023). However, further examination at *K* = 3 in the admixture analysis revealed that the genetic background of the Lari gamecocks exhibited similarities with RJF, consistent with findings from a previous study that highlighted the proximity of Chinese gamecock breeds to RJF (Zhang et al. 2020). This suggests that gamecocks have retained a substantial portion of their genetic heritage from their ancestors, further highlighting their unique genetic characteristics. It is important to acknowledge that game chickens are selectively bred for their aggressive behaviour and fighting abilities in the sport of cockfighting, and they are often kept purebred without crossbreeding with other indigenous chickens.

We also observed that the WLH line demonstrated a more distant genetic distance from RJF compared to the ARI line, an indication of WLH to have undergone significant genetic changes over the past century following strong artificial selection for high egg production. Furthermore, we noticed some level of genetic admixture of the IFN, TBZ, SRY and MHD ecotypes with ARI, which was developed in northern Iran, where local farmers often crossbred these ecotypes with the ARI birds to enhance the meat characteristics of indigenous chickens.

Within‐population genetic diversity is influenced by various factors, including migration, genetic drift and selection, all of which impact on allele distribution (Allendorf, Luikart, and Aitken 2007). In our study, the lowest genetic diversity belongs to commercial lines. These lines have been developed independently by different breeding companies, focussing on specific traits over the past century. Intense selection in the commercial lines has resulted in the fixation of certain alleles associated with desired traits, leading to a reduction in genetic diversity compared to their wild ancestors or indigenous chicken populations that have experienced limited artificial selection (Groeneveld et al. 2010; Qanbari et al. 2019).

Among indigenous ecotypes, SRY revealed a higher level of genetic diversity compared to RJF. This finding is consistent with the estimations by Zhi et al. (2023), who found the genetic diversity of RJF to be lower than that observed in most indigenous populations. The significant genetic diversity in the SRY population suggests a greater potential for adaptation and resilience in the face of environmental challenges. Overall, we found heterozygosity values close to 0.15 in indigenous chicken populations, which were lower than those observed for Tibetan and multiple indigenous chicken breeds (0.3) (Tan et al. 2024; Yuan et al. 2022) and Chinese indigenous chickens (0.22) (Sun et al. 2022; Zhang et al. 2018). This discrepancy may indicate potential inbreeding in Iranian chicken ecotypes. Given that genetic diversity in indigenous chicken populations is essential for their overall health and adaptability, conservation efforts should prioritise the preservation of viable populations of the indigenous ecotype. This can be achieved through the creation of protected areas, conservation breeding programs, and the adoption of sustainable management practices.

### 4.2 Runs of Homozygosity and Genomic Inbreeding

Observational genotype‐counting algorithms and model‐based algorithms are the two primary approaches used to identify ROH in genetic studies (Pemberton et al. 2012; Purcell et al. 2007). An example of an observational genotype‐counting algorithm is the PLINK software, which has shown superior performance in detecting ROH compared to model‐based algorithms like Germline and Beagle (Howrigan, Simonson, and Keller 2011). In this study, ROH calling was performed using Plink software. Due to the relatively low coverage of whole genome sequence data to detect ROH, which may result in a higher error rate compared to array data (Ceballos et al. 2018), we adjusted the parameters based on Ceballos et al. (2018). However, we chose a sliding window length of 100 to capture more detailed information about the distribution of ROH in the genome and potentially identify smaller ROH fragments that could be biologically relevant. In populations with lower genetic diversity or higher levels of inbreeding, smaller ROH fragments may still be informative and relevant.

The distribution of ROH in a genome can vary due to various factors, including breeding practices, selection for specific traits and the founder effect. For instance, in commercial breeds, rigorous selective breeding programs are implemented to enhance traits like growth rate, egg production and meat quality based on mating closely related individuals to fix desired alleles, which can result in an elevated likelihood of homozygosity and longer ROH in the genome (Rebelato and Caetano 2018). In this study, the commercial breeds exhibited the highest ROH coverage, with 229 Mb for ARI and 257 Mb for WLH, in line with the findings claimed by Sun et al. (2022). These results were further supported by the high *F*_ROH_ values in these breeds. On the other hand, RJF encompasses the lowest ROH coverage among all the studied populations, which agrees with the observations from previous studies (Sun et al. 2022; Talebi et al. 2020). The average length of ROH in indigenous chicken populations was obtained 134 Mb, significantly higher than the result of 55.53 Mb observed in the analysis of Chinese chickens (Tian et al. 2023). The varying patterns of ROH length can elucidate the differences between breed origin and recent management practices.

Across all populations, the predominant length of ROH segments was found to be shorter than 1 Mb, aligning with previous research using whole genome‐sequencing data (Sun et al. 2022; Tan et al. 2024). A higher proportion of shorter ROH suggests that these populations may have been initially originated from small founding populations, but they have not been significantly impacted by recent inbreeding (Brito et al. 2017).

Our analysis of longer ROH category (> 1 Mb) in commercial chicken breeds revealed that the ARI breed carried a larger proportion of medium and long ROH (> 1 Mb) than the WHL line, which is likely attributed to a higher level of recent inbreeding resulting from selective close mating for specific traits such as rapid growth and breast muscle size.

Our study of ROH segment distribution across autosomes of various chicken populations showed that GGA16 consistently had the lowest coverage of ROH, both indigenous and commercial chickens. Similar to previous research on Ugandan ecotypes (Fleming et al. (2016), RJF also exhibited no ROH on GGA16. This lack of ROH is indicative of high genetic diversity, likely driven by the presence of highly polymorphic and polygenic Major Histocompatibility Complex (MHC) genes located on this chromosome (Miller & Taylor Jr 2016).

ROH‐based inbreeding estimates are known for their accuracy, precision, and consistency across populations (Peripolli et al. 2017). Several studies have highlighted the superiority of ROH‐based inbreeding (*F*_ROH_) over pedigree‐ and marker‐based estimates in quantifying inbreeding levels (Ferencakovic et al. 2011; Keller, Visscher, and Goddard 2011; Mastrangelo et al. 2016; Zhang et al. 2015). The *F*_ROH_ offers several advantages. Firstly, it is not dependent on pedigree information, which can be limited or inaccurate in some populations. Secondly, ROH analysis provides a more comprehensive assessment of inbreeding history. Thirdly, *F*_ROH_ is a robust measure of recent inbreeding events, as it is not influenced by allele frequencies. This contrasts with methods like *F*_GRM_, *F*_HOM_ and *F*_UNI_, which rely on estimated allele frequencies and may introduce biases in inbreeding level estimations (Zhang et al. 2015). Moreover, *F*_ROH_ can be calculated across all autosomes, providing a more detailed and informative assessment of inbreeding patterns (McQuillan et al. 2008).

Our investigation into the contribution of different chromosomes to genomic inbreeding coefficients revealed that GGA22 displayed a relatively higher contribution across all populations. This observation suggests that the presence of specific genes or functional elements on GGA22 may confer selective advantages, leading to a higher frequency of homozygous individuals carrying these beneficial variants.

As expected, the commercial lines exhibited the highest *F*_ROH_ values, while RJF showed the lowest, consistent with the findings of (Zhang et al. 2020). This pattern aligns with the expectation that intensive selection in commercial lines can lead to high levels of inbreeding. Indigenous Iranian chickens displayed a range of inbreeding levels from 0.11 to 0.17. The observed differences in inbreeding levels are likely influenced by the distinct selection pressures exerted on each ecotype.

It is important to note that estimating F (inbreeding) from ROH has its own limitations. It requires high‐quality genomic data and accurate identification of ROH regions (Ceballos et al. 2018; Purfield et al. 2012). Additionally, the interpretation of ROH results can be intricate due to potential influences from factors such as population structure and selection (Forutan et al. 2018; Peripolli et al. 2017).

### 4.3 Putative Signatures of Selection for Disease Resistance

Positive selection can lead to increases in the frequency of advantageous alleles and then the probability of homozygosity in certain genomic regions towards ROH islands over time. If the ROH islands are present in a significant proportion (e.g., >50%) in the population, they can be indicative of selective pressure (Nothnagel et al. 2010). In this study, we considered the ROH islands shared by > 70% of the birds in each population as putative selection signatures. Nevertheless, it is important to exercise caution that the short ROH islands can be influenced by various evolutionary processes and demographic events. For instance, some of such short ROH islands may represent identical‐by‐state (IBS) regions, where individuals share the same homozygous genotypes by chance due to their recent common ancestry (Mastrangelo et al. 2017; Purfield et al. 2017).

Chickens raised in rural areas are indeed subjected to a wide range of environmental challenges and successful survival over such challenges requires their strong and rapidly evolved adaptation (Fleming et al. 2017). Several studies have consistently demonstrated that certain ROH islands can harbour candidate genes associated with immune response and adaptation to such challenges (Marchesi et al. 2018; Mastrangelo et al. 2017; Rostamzadeh Mahdabi et al. 2021). We identified significant enrichments (*p* ≤ 0.05) in some genes mapped in the ROH islands in most populations. For instance, the cytokine‐mediated signalling pathway (GO: 0019221) (*p* ≤ 0.05) was enriched in five ecotypes (IFN, SYZ, TBZ, ZAH and LR‐IR) and RJF. The *IL22RA1* and *IFNLR1* genes on GGA23 were involved in this pathway. *IL22RA1* is known for its role in disease resistance (Xiong, Liu, and Rao 2023) as it encodes a receptor for IL‐22, an important cytokine in host defence and inflammatory responses (Gaudino et al. 2021). *IFNLR1* is involved in the antiviral response in chickens (Reuter et al. 2014). In the TBZ ecotype, *IFNLR1* was annotated in the GO term of defence response to viruses (GO: 0051607). This gene was also identified in a ROH island of yellow‐feathered chickens (Weng et al. 2022). We identified the* STAT1* gene on GGA7, which was annotated in several GO terms associated with molecular function, cellular content, biological process and KEGG pathways, including cytokine‐mediated signalling (GO: 0019221) (enriched in IFN, LR‐IR, SYZ, TBZ and ZAH) and Influenza A (gga05164) (enriched in SYZ). *STAT1* has been shown to contribute to disease resistance (Klees et al. 2022) by transmitting signals from interferons to activate the immune response and create an antiviral environment in cells (Tolomeo, Cavalli, and Cascio 2022). Additionally, increased *STAT1* phosphorylation and IFN‐stimulated *IFITM3* expression through the overexpression of chMertk, a membrane protein involved in regulating innate immune response, resulted in a significant reduction in Newcastle disease virus titers (Tan et al. 2023).

The IFN, LR‐AF and SYZ populations were found to have enrichment of GO terms related to biological processes such as defence response to viruses (GO: 0051607) and inflammatory response (GO: 0006954), as well as the KEGG pathway associated with Influenza A (gga05164). These populations contain the *TICAM1* gene located on GGA28, which functions within signalling pathways mediated by *TLR3* and *TLR4*. This facilitates the activation of regulatory factors such as interferons (IFNs) and nuclear factor kappa B (NF‐kB) (Seya et al. 2005; Wu et al. 2011), leading to the production of type I IFNs and pro‐inflammatory cytokines for the elimination of invading pathogens (Kannaki et al. 2010).

The genomic regions on GGA4 that were identified for the SYZ, SRY and LR‐AF populations contained candidate genes associated with local adaptation, such as *NFKB1* and *TLR2*. The *NFKB1* gene, located on GGA4, which plays a significant role in the immune system, was significantly enriched in the KEGG pathway of Influenza A (gga05164). *NFKB* transcription factors are implicated in immune responses (Hayden and Ghosh 2008). Activation of Toll‐like receptors (TLR) occurs when they bind to pathogen‐associated molecular patterns (PAMPs), initiating cascades that rely on MAPK or nuclear factor kappa B (NFkB) factors. This activation leads to a pro‐inflammatory response and the secretion of antibacterial substances like *β*‐defensins and cytokines (Kogut et al. 2006). Walugembe et al. (2019) reported that the *NFKB1* gene may have a role in the survival of Sri Lankan ecotypes in the hot and humid tropical conditions of Sri Lanka and it has been identified as a candidate gene under selection in these ecotypes. Another gene, *TLR2*, has been shown to have a direct impact on the immune response in poultry hosts (Kannaki et al. 2010; Lu et al. 2009). When *TLR2* is activated by various pathogen‐associated molecular patterns (PAMPs) from viruses, bacteria, fungi or parasites, inflammatory responses are triggered (Hausmann et al. 2002). It has been demonstrated that chicken *TLR2* can provide protection against avian influenza and Newcastle disease viruses, as well as Gramme‐negative bacteria like Salmonella and *Escherichia coli* (Paul et al. 2013). Indigenous chicken ecotypes, compared to industrially‐raised poultry maintained under controlled conditions, are more exposure to pathogenic diseases. Therefore, it is expected that they would exhibit better performance in terms of the immune system and have undergone selection for genes related to resistance against pathogenic factors (Sukhija et al. 2023).

### 4.4 Putative Signatures of Selection for Heat Tolerance and Oxidative Stress

The increase in global average temperature over the past century has likely resulted in the development of village chickens with genetic traits that confer higher heat tolerance. This selective pressure may have driven the evolution of heat‐resistant ecotypes. Heat stress can compromise the immune system, making chickens more susceptible to diseases and increasing mortality rates (Hirakawa et al. 2020; Sukhija et al. 2023). Heat shock proteins (HSPs) and heat shock factors (HSFs) in chickens play a fundamental role in cellular physiology and protection against various stressors, including heat stress (Balakrishnan, Ramiah, and Zulkifli 2023). The *HSF4* gene is one of the important stress proteins as it regulates the function of HSPs (Farhan, Arabdin, and Khan 2021). This gene was identified in the SYZ and LR‐IR ecotypes, which likely contributes to their ability to tolerate and adapt to the high temperatures prevalent in Shiraz province. We also discovered two other genes, *BDNF* and *OSGIN1*, that overlap with ROH islands, which are associated with heat tolerance and oxidative stress. The brain‐derived neurotrophic factor (*BDNF*) gene, found in seven indigenous ecotypes, plays a role in the response to heat stress. *BDNF*, a neurotrophin, is involved in induced adaptation in chicks (Katz and Meiri 2006). The expression of early heat‐induced stress response genes, such as *BDNF*, serves as a protective mechanism to prevent cellular damage in various tissues (Goel, Ncho, and Choi 2021). Heat stress is widely recognised as one of the most challenging environmental stressors and a major contributor to systemic oxidative stress (Altan et al. 2003; Mishra and Jha 2019). The oxidative stress‐induced growth inhibitor 1 (*OSGIN1*) gene, identified as a selection signature in all native ecotypes, contributes to the reduction of oxidative stress (Brennan et al. 2017; Ott et al. 2002). It has also been detected in the ROH mapping of Fayoumi chickens, where its homozygous genotype is believed to contribute to the Fayoumi ecotype's response to oxidative stress by regulating cell death and apoptosis in (Elbeltagy et al. 2019; Ott et al. 2002). Managing oxidative stress is crucial as it can negatively affect overall performance and impact the quality of meat and eggs (Estévez 2015; Mishra and Jha 2019). Oxidative stress in poultry can be caused by various factors, including nutritional imbalances, environmental heat stress and pathological conditions (Akbarian et al. 2016; Lin, Decuypere, and Buyse 2006). The *OSGIN1* gene in Iranian indigenous ecotypes may play an important role in mediating the cellular response to heat stress and other environmental stressors by regulating key pathways involved in stress adaptation.

### 4.5 Putative Signatures of Selection for Production and Reproduction

Among the Iranian chicken ecotypes, the Lari chickens are known for their high weight (3–4 kg) and are specifically bred for their game fighting abilities. They have tall stature and strong muscles, which contribute to their agility and power during fights. Within the identified ROH islands on GGA2 in the Lari ecotype, the *MEOX2* gene was annotated. This gene is known to play a role in the development of skeletal muscle, bone, and vasculature (Kokotović et al. 2022). Studies on mice have shown that *MEOX2* can influence muscle size and mass (Mankoo et al. 1999; Otto et al. 2010). Furthermore, *MEOX2* was also involved in limb development, as indicated by its association with the GO term ‘limb development’ (GO: 0060173).

We encountered two important genes, *CEBPB* and *CDS2*, in the ROH islands of all native chicken ecotypes. *CEBPB* is believed to have a role in chicken growth (Chen et al. 2015), specifically in regulating glycogen levels in the skeletal muscle of chickens through the PKA‐cAMP pathway (Sibut et al. 2011). It also regulates multiple genes stimulated by growth hormone (GH) (Cui et al. 2011), which further contributes to chicken growth and development. *CDS2* is involved in glycerophospholipid metabolism (46) and is expressed in multiple fat tissues in chickens (Xu et al. 2022) and is also associated with fat deposition in sheep (Bakhtiarizadeh and Alamouti 2020).

Indigenous chickens are mostly managed under a scavenging system that offers varied diets to impart unique flavour and rich taste of chicken meat, which is strongly influenced by level of lysophosphatidylcholine (LPC). The *PLA2G4A* gene is involved in the biosynthesis pathway of LPC (Gai et al. 2023) and also has an impact on lipid metabolism, triglyceride content of adipose tissue, and other metabolic pathways (Hamill et al. 2013; Yi et al. 2015). It is worth noting that *PLA2G4A* is mapped to a ROH island of seven indigenous ecotypes included in this study.

Interestingly, we also observed that some genes in the ROH islands of indigenous chickens were over‐represented in pathways related to reproduction, for example, the *GnRH* signalling pathway (gga04912, *p* < 0.01). It is known that *GnRH1* serves as a key regulator of reproductive and egg production traits (Bhattacharya et al. (2019) in various chicken breeds by stimulating the secretion of LH and FSH hormones, which in turn govern the development of reproductive organs, gamete production and the initiation of reproductive processes (Bédécarrats, Shimizu, and Guémené 2006; Marques et al. 2000). *GnRH1* could potentially be used as a marker in breeding programs of native chickens (Badi, Ayied, and Al‐Salhie 2023).

Overall, these results suggested that the genes within the ROH islands might be relevant to heat tolerance, immunity, production and reproduction traits, and defence against viral infections in chickens. These characteristics were well aligned with the ability of local chickens to withstand the challenging hot and arid climate of Iran.

## 5 Conclusion

This study delved into the population genetic structure and diversity as well as the selection signatures of Iranian indigenous chicken ecotypes compared to commercial breeds and RJF. Our findings showed that gamecock populations shared a similar genetic background with RJF, while the WLH line displayed a relatively distant genetic relationship with RJF compared to the ARI line. Among the indigenous ecotypes, the SRY had the highest genetic diversity. The identification of ROH islands associated with adaptive and production‐related traits in this study can assist in selecting birds with desirable genetic variants, ultimately contributing to the genetic improvement and sustainability of indigenous chicken genetic resources. While the results obtained from the current sample size provide valuable insights, there is a need to consider the potential limitations of generalisability and statistical power that come with a smaller sample size. In conclusion, further studies are needed to validate our findings using a larger sample and size whole genome sequencing with greater depth. Conducting replication studies with increased sample sizes and more comprehensive genetic data will enhance the reliability and generalisability of the results, ultimately contributing to a deeper understanding of genetic variations and traits in the studied chicken populations.

## Author Contributions

**Elaheh Rostamzadeh Mahdabi**: formal analysis, methodology, visualisation, writing–original draft. **Ali Esmailizadeh**: funding acquisition, project administration, supervision, visualisation, writing–review and editing. **Jianlin Han**: conceptualisation, data curation, investigation, writing–review and editing. **Ming‐Shan** **Wang**: conceptualisation, data curation, funding acquisition, methodology, writing–review and editing.

## Conflicts of Interest

The authors declare no conflicts of interest.

### Peer Review

The peer review history for this article is available at https://publons.com/publon/10.1002/vms3.70074.

## Supporting information



Supporting Information

Supporting Information

Supporting Information

Supporting Information

Supporting Information

Supporting Information

## Data Availability

The datasets used for the current study were from Wang et al. (2020, doi: 10.1038/s41422‐020‐0349‐y). The raw sequencing data, alignment BAM files and genotypes (VCF) are available in the ChickenSD database (http://bigd.big.ac.cn/chickensd/) and Chicken2K (http://chicken.ynau.edu.cn).
