# Supplementary material for: Comparative Analysis of Runs of Homozygosity Islands in Indigenous and Commercial Chickens Revealed Candidate Loci for Disease Resistance and Production Traits
Source: Vet Med Sci. 2024 Dec 10;11(1):e70074. doi: 10.1002/vms3.70074 (PMC11629026; doi:10.1002/vms3.70074)
Supplement: Supplementary file 1 — Supporting Information [file VMS3-11-e70074-s006.docx]

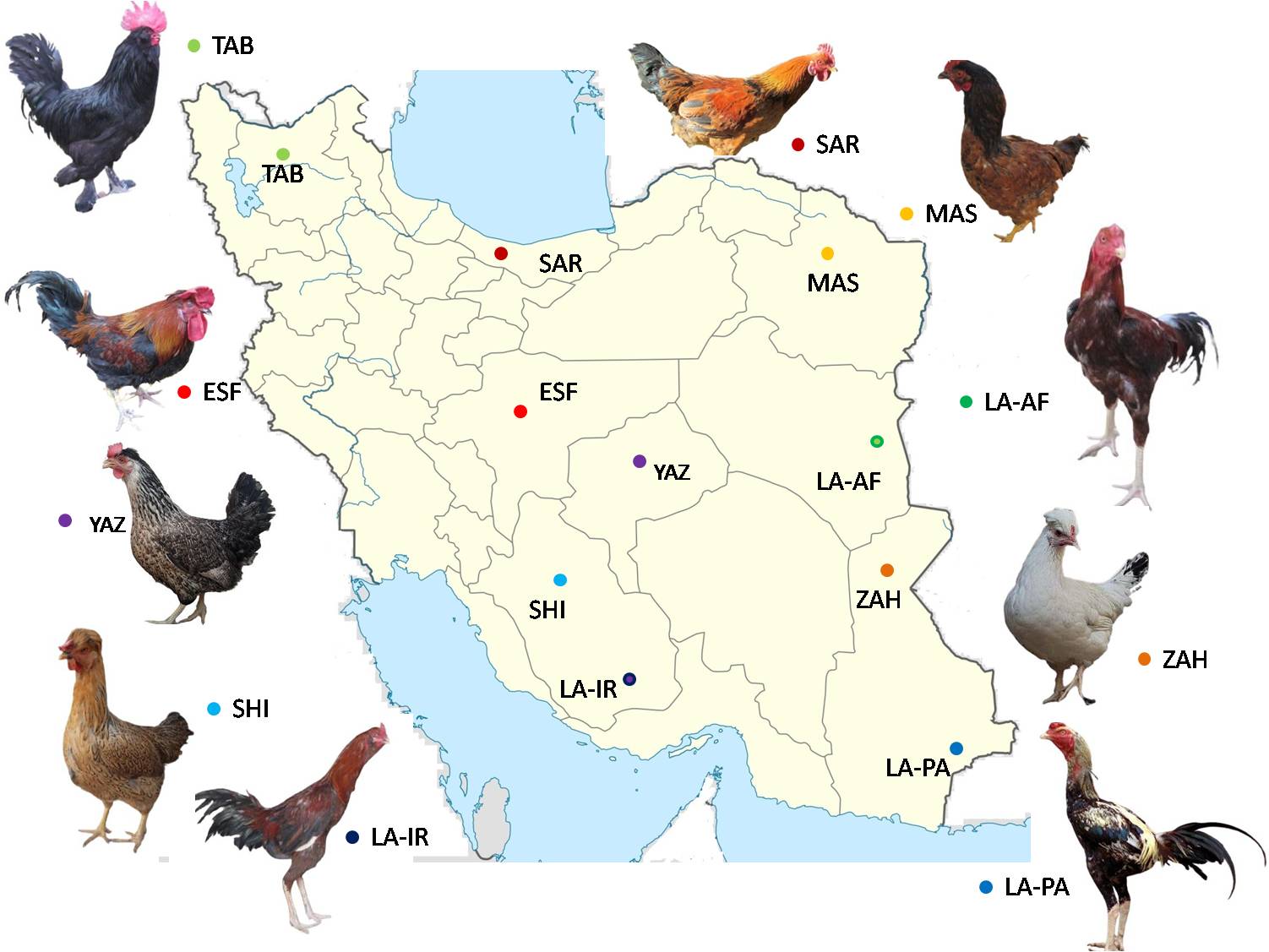


Fig S1. Geographic distribution and phenotypic diversity of Iranian indigenous chicken breeds
