# Supplementary material for: Comparative Analysis of Runs of Homozygosity Islands in Indigenous and Commercial Chickens Revealed Candidate Loci for Disease Resistance and Production Traits
Source: Vet Med Sci. 2024 Dec 10;11(1):e70074. doi: 10.1002/vms3.70074 (PMC11629026; doi:10.1002/vms3.70074)
Supplement: Supplementary file 3 — Supporting Information [file VMS3-11-e70074-s005.docx]

Table S2. Mean number of ROH per individual and inbreeding coefficients (*F*_ROH_) estimated in different length categories

| Breed | Mean number of ROH |  | *F*_ROH_ |  |
| --- | --- | --- | --- | --- |
|  |  | 0.1-1 Mb | 1-2 Mb | >2 Mb |
| IFN | 511 | 0.126 | 0.016 | 0.009 |
| MHD | 470 | 0.108 | 0.005 | 0 |
| SRY | 468 | 0.112 | 0.013 | 0.007 |
| SYZ | 532 | 0.131 | 0.021 | 0.005 |
| TBZ | 461 | 0.104 | 0.012 | 0.007 |
| AZD | 588 | 0.138 | 0.013 | 0.004 |
| ZAH | 505 | 0.119 | 0.022 | 0.011 |
| LR-AF | 517 | 0.128 | 0.022 | 0.01 |
| LR-IR | 563 | 0.148 | 0.025 | 0.007 |
| LR-PK | 447 | 0.103 | 0.006 | 0 |
| ARI | 794 | 0.222 | 0.045 | 0.012 |
| WLH | 856 | 0.216 | 0.028 | 0.005 |
| RJF | 419 | 0.099 | 0.007 | 0.002 |
